# The benefits of sensation on the experience of a hand: A qualitative case series

**DOI:** 10.1371/journal.pone.0211469

**Published:** 2019-01-31

**Authors:** Emily L. Graczyk, Anisha Gill, Dustin J. Tyler, Linda J. Resnik

**Affiliations:** 1 Department of Biomedical Engineering, Case Western Reserve University, Cleveland, Ohio, United States of America; 2 Louis Stokes Cleveland Veterans Affairs Medical Center, Cleveland, Ohio, United States of America; 3 Providence Veterans Affairs Medical Center, Providence, Rhode Island, United States of America; 4 Department of Health Services, Policy, and Practice, Brown University, Providence, Rhode Island, United States of America; University of Rome, ITALY

## Abstract

**Background:**

The experience of upper limb loss involves loss of both functional capabilities and the sensory connection of a hand. Research studies to restore sensation to persons with upper limb loss with neural interfaces typically measure outcomes through standardized functional tests or quantitative surveys. However, these types of metrics cannot fully capture the personal experience of living with limb loss or the impact of sensory restoration on this experience. Qualitative studies can demonstrate the viewpoints and priorities of specific persons or groups and reveal the underlying conceptual structure of various aspects of their experiences.

**Methods and findings:**

Following a home use trial of a neural-connected, sensory-enabled prosthesis, two persons with upper limb loss were interviewed about their experiences using the sensory restoration system in unsupervised, unconstrained settings. We used grounded theory methodology to examine their experiences, perspectives, and opinions about the sensory restoration system. We then developed a model to describe the impact of sensation on the experience of a hand for persons with upper limb loss.

**Conclusions:**

The experience of sensation was complex and included concepts such as the naturalness of the experience, sensation modality, and the usefulness of the sensory information. Sensation was critical for outcome acceptance, and contributed to prosthesis embodiment, confidence, reduced focus and attention for using the prosthesis, and social interactions. Embodiment, confidence, and social interactions were also key determinants of outcome acceptance. This model provides a unified framework to study and understand the impact of sensation on the experience of limb loss and to understand outcome acceptance following upper limb loss more broadly.

## Introduction

Following an upper limb amputation, many persons utilize a prosthesis to aid them in daily life. However, approximately 21% of adults with upper limb loss do not use prostheses at all, in part because they feel that current prosthetic devices do not meet their needs [1]. This may be partially due to the fact that commercially available prostheses provide mobility and function, but do not restore the sensory capabilities of the hand. Approximately 85% of prosthesis rejecters identify the lack of sensory feedback as a factor in their decision not to wear a prosthesis [2,3]. Without sensory feedback to regulate grip force and determine material properties of objects, myoelectric prosthesis users must rely to a larger extent on visual and auditory feedback from observing the prosthesis, resulting in functional deficits [4–10]. In addition, sensory feedback aids in prosthesis incorporation into the body, and may contribute to the psychosocial experience of having a hand [11,12].

Multiple research groups are working to augment prosthetic hands with naturalistic sensation, delivered via electrical stimulation applied directly to the peripheral nerves [13–18], spinal cord [19], or cortex [20]. Electrical stimulation can produce tactile and proprioceptive sensations referred to the phantom hand and limb. These groups have shown that restored sensation can improve the functionality of prosthetic hands, improve prosthetic incorporation, increase usage of the prosthesis, and have other beneficial psychosocial outcomes [14,15,21,22]. However, it is not known if these improvements on quantitative outcome measures will translate into better user attitudes and higher prosthesis satisfaction.

Although prior research demonstrates that persons in the upper limb community strongly desire sensory enabled prostheses, the anticipated benefits of having this sensation are less clear [1,10]. Furthermore, we do not know to what extent upper limb amputees will be willing to undergo the risks associated with surgically invasive interfaces in order to achieve sensory restoration, which may itself be less than ideal. A recent study found that while 86% of amputees would be willing to try non-invasive myoelectric control, 64% would be willing to try peripheral nerve interfaces for control, and only 38% would be willing to try cortical interfaces for control [23]. In order to understand the cost-benefit balance that amputees may employ when making a decision about sensory restoration, we must first understand what the perceived benefits of sensation are from their own perspectives.

Qualitative research of persons with upper limb loss can provide a meaningful view into the values, perspectives, opinions, and priorities of this community in a way that structured surveys and standardized metrics cannot. Qualitative analysis has been utilized to understand the psychosocial adjustment process to lower limb amputation in diabetics [24], the psychosocial adjustment to traumatic amputation in women [25], the holistic impact of a novel terminal device on upper limb amputees [26], the experience of successful prosthesis users [27], the experience of living with an osseointegrated prosthesis [11], and the experience of living with limb loss [28,29]. However, to our knowledge, the role of sensation in the experience of limb loss has not been investigated using qualitative methods.

The general amputee population cannot accurately assess the impact of restored sensation, since they have never actually had the experience of using a sensorized prosthesis. While these persons can imagine and attempt to predict the ways that having sensation would impact their daily lives, their predictions are not grounded in actual experience [30]. It would be better to assess the impact of sensation in research participants who have experienced temporary restoration of sensation within a laboratory setting. Yet, these types of assessments are limited as well, given that these persons have only limited experience due to constraints of using their sensorized prostheses in controlled settings and under supervision. Given the lack of experience with a sensorized prosthesis in a naturalistic environment, the perceived impact of having sensation may be incomplete or even faulty.

In order to understand the impact of sensation on daily life, our group conducted a take home trial of a sensory restoration system with two upper limb amputee participants [22]. The purpose of this study was to understand the impact of sensory restoration on functional performance, prosthesis usage, and psychosocial outcomes. In this study, the participants received tactile and proprioceptive sensations through electrical stimulation of their peripheral nerves when they used a sensorized prosthesis to interact with objects or people. Participants wore the system at home for a week with free choice in how and when to use it and without daily researcher supervision. Although numerous functional measures and surveys were administered as part of this study, we found that they did not fully capture the participants’ opinions and perspectives about their experiences of sensory restoration. Because of this, in the present study, we performed a qualitative analysis of data from participant interviews following the take home trial of the sensory restoration system. Our purpose was to elucidate the opinions and perspectives of these participants based on their lived experiences of having a prosthetic hand that could feel. In a way never before possible, this study provided the opportunity for an in-depth exploration of the impact of having a sensory restoration myoelectric prosthesis from the perspective of persons with upper limb loss.

## Methods

### Participants

Two persons with right, unilateral, upper limb amputation participated in this study. Participant 1 (P1), a 49 year old male, had a right transradial amputation in 2009 due to traumatic injury. Participant 2 (P2), a 50 year old male, had a right transradial amputation in 2004 due to traumatic injury. Both participants were right hand dominant prior to injury. Participant 1 was implanted with 8-channel Flat Interface Nerve Electrodes (FINEs) around his median and ulnar nerves and a 4-channel Case Western Reserve University (CWRU) spiral cuff around his radial nerve in 2012. Participant 2 was implanted with 8-channel FINEs around his median and radial nerves in 2013. Subject 1’s implants were placed in the forearm, and subject 2’s implants were placed in the mid-upper arm [15]. Application of electrical stimulation pulse trains to FINEs implanted on the residual peripheral nerves results in evoked tactile and proprioceptive sensations perceived to be located on the missing hand and fingers.

Subject 1 had 13 years of education and Subject 2 had 12 years of education. Both subjects were users of myoelectric prostheses prior to enrollment in the take home study. Subject 1 had been using a myoelectric prosthesis regularly for 4 years before the take home study. Subject 2 had been using a myoelectric prosthesis regularly for 3.5 years before the take home study. Both report using a myoelectric prosthesis as their primary prosthetic device, and wear it most of the day (8–12 hours/day). The home use study took place between months 41 to 42 post-implant for both subjects.

**Fig 1:**
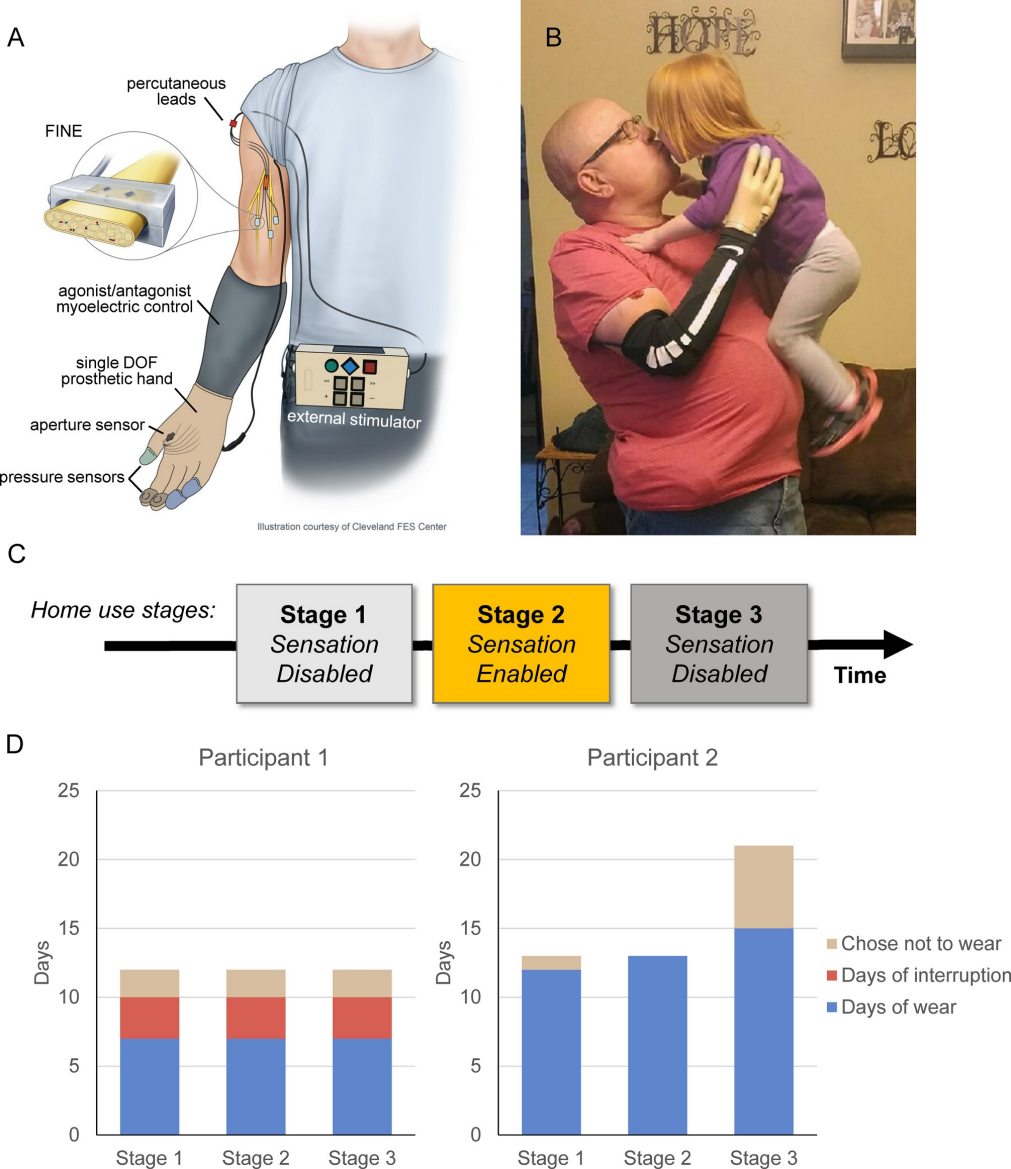
Take home sensory restoration system and study design. A) Schematic diagram of the take home system demonstrating system components and connections. B) A participant uses the sensory restoration system to embrace a child. C) Timeline of the study. Participants used the prosthesis system without sensory feedback in stages 1 and 3 and with sensory feedback enabled in stage 2. The participants came to the lab for testing sessions before the start of the study and after each stage. D) System wear-time per stage for participants 1 and 2.

All study devices and procedures were reviewed and approved by the U.S. Food and Drug Administration Investigational Device Exemption, the Cleveland Department of Veterans Affairs Medical Center Institutional Review Board, and the Department of the Navy Human Research Protection Program. All study procedures and experiments were performed in accordance with relevant guidelines and regulations of these institutions. Written informed consent was obtained from both subjects. The individual in this manuscript has given written informed consent (as outlined in the PLOS consent form) to publish the photograph and case details presented in Fig 1.

### iSens take home system

Participants wore a sensory restoration system in their homes and communities (Fig 1A and 1B). The system consisted of a myoelectric prosthetic socket, custom-sensorized Ottobock VariPlus Speed prosthetic hand, nerve stimulator, and several cables for connections between system components. The VariPlus Speed prosthetic hand is a single degree-of-freedom electric hand, which performs an open/close movement which resembles a power grasp. Participants wore their own prosthetic sockets with standard agonist/antagonist control. With this control scheme, electromyography electrodes in the prosthetic socket pick up electrical activity from the contractions of the user’s residual forearm muscles. The user contracts the forearm flexors to close the hand and contracts the forearm extensors to open the hand, where prosthesis speed is controlled by the magnitude of the muscle contraction. The augmented VariPlus Speed prosthetic hand included custom pressure sensors on the tips of the thumb, index, and middle fingers. The custom sensors consisted of a force sensitive resistor (FSR) embedded in silicone and covered with finger cots. The silicone coating conveyed forces from most common orientations of object interaction with the prosthesis and the cots protected the sensors during daily use. A custom aperture sensor that measured the width of prosthetic opening was installed underneath the prosthetic cosmetic cover. The sensor information was sent via a cable to the nerve stimulator. The stimulator transformed the information into an appropriate nerve stimulation pulse train and transmitted it through a second external cable to the participant’s percutaneous leads and then to the FINEs implanted on the residual somatosensory nerves. For both subjects, the four sensors on the prosthetic hand (3 for fingertip pressures and 1 for hand aperture) were mapped to four stimulation channels in the subject’s 8-channel FINE on their median nerve. The stimulation channels delivered biphasic, charge-balanced, cathode-first stimulation pulse trains to the median nerve. Touch on a fingertip sensor resulted in perceived sensation on the participant’s phantom hand, collocated with the position of the sensor on the prosthetic hand. For example, touch on the index sensor yielded sensation on the perceived index finger of the phantom. The aperture sensor corresponded to a tactile percept on the palm for Subject 1 and a proprioceptive sensation of hand closing for Subject 2. Changes in pressure on the fingertip sensors or changes in the width of the prosthetic aperture were translated into changes in sensation intensity using pulse frequency [18,22].

### Study design

The overall home study was a quasi-experimental study that utilized a cross-over design with three stages (Fig 1C). At the start of the study, participants visited the laboratory for training on the system and in-lab testing. The first stage, designed to provide time for the participants to acclimate to wearing the system and using the new prosthetic terminal device, was two weeks in length. Participants wore the sensorized terminal device and used their normal myoelectric controls, but had the sensory stimulation turned off. During the second stage, which was intended to be a week in length, participants wore the sensory restoration system with the stimulation turned on. Finally, during the third stage, which was two weeks in length, the participants wore the same prosthesis and system with the stimulation turned off. Participants visited the laboratory at the end of each Stage for in-lab testing and activation/deactivation of the sensory feedback as appropriate.

Participant 1 wore the system in each Stage for a total of 7 days (Fig 1D). The actual length of each Stage exceeded 7 days because there were several instances of device malfunction where the system hardware was unusable for 3–4 days while it was being repaired. Participant 2 wore the system for 12, 13, and 15 days in Stages 1, 2, and 3, respectively. In Stage 2, Participant 2 wore the system with sensation for 12 consecutive days. In this case, Stage 2 was extended to accommodate the participant’s travel schedule. Stage 3 was also extended for Participant 2 due to an illness that prevented the participant from travelling to the laboratory (Fig 1D).

Each day, the participants independently donned the system and made all cabling connections. On days in which sensation was enabled, participants calibrated their stimulation settings to ensure that the perceived sensations were suprathreshold and comfortable. Participants could modify the stimulation settings whenever they chose during Stage 2.

### Data collection

During the home study, participants completed daily surveys about their experiences and sensations. In laboratory testing sessions, they completed additional surveys related to perceptions of prosthesis use and performed standardized functional tasks with the prosthesis. The quantitative portions of this data have been reported previously [22].

The present manuscript analyses the qualitative data collected throughout the study, which includes participants’ written responses to free response questions on daily diaries and in-lab surveys and verbal responses to an unstructured interview with researchers in the laboratory setting. Participants completed daily diaries in which they were encouraged to write about the prosthesis, the sensations evoked by the sensory stimulation, and any other comments about their experiences (see S2 Appendix for daily diary free response questions). At the beginning of the study and after each stage, participants completed testing sessions in the laboratory environment in which they performed standardized functional tasks and described their experiences with these tasks using a written free response form (see S2 Appendix). At the conclusion of Stage 2, they participated in an unstructured interview with research staff. Interview questions focused on the sensory experience and sensory restoration system (see S2 Appendix), with additional probing by the interviewer to clarify or expand comments. Interviews were audio recorded and transcribed.

### Data analysis

This qualitative study aimed to develop a complex, holistic understanding of participants’ experience with the sensory restoration system (SRS). Data analysis was performed by three investigators using a grounded theory approach with constant comparison methods [31]. The intent of grounded theory is to generate a framework explaining the inter-relationships of concepts related to a particular situation [32,33]. This approach is appropriate when studying a new phenomenon where the relationships between concepts are poorly understood [34].

Three of the investigators (EG, LR, AG) independently read the interview transcripts and written responses from the diaries and performed line by line open coding of key concepts they observed within the data [33]. NVivo 11 software was used to organize data (NVivo qualitative data analysis Software; QSR International Pty Ltd. Version 11, 2017). NVivo stores source documents in a central repository, and enables linking of segments of text to user-specified labels (nodes). NVivo enables users to group text segments by node, and can generate statistics and visualizations of coded content.

Initial codes were discussed and compared, and iteratively fine-tuned through consensus of the three data analysts. The codes were organized into several over-arching categories and further divided into sub-categories. Complete descriptions of each code were generated and supported by rich text exemplars. These descriptions were then reviewed by the three analysts, which resulted in iterative refinement in the coding categories, their descriptions, and organizational structure. Repeated refinement of the coding structure led to the final coding structure shown in Table 1. Memos of the analytic process were written to serve as an audit trail of decisions made during data analyses.

**Table 1:** Coding structure. Left column indicates category, middle column indicates sub-category, and right column describes the content coded within each sub-category. White rows were included in the model; grey rows were not.

	Sub-categories	Description
**Return to Normalcy**	Naturalness of experience	Statements about the naturalness of the participant’s experiences (or lack of naturalness), including sensation being comparable to the intact hand, and the sensory experience being integrated into their daily routines and decision making processes
	Embodiment	Comments indicating the presence (or absence) of self-attribution or ownership of the prosthesis, how the system affected participants’ perceptions of wholeness, and that the experience of the hand remained after stimulation was off
	Interaction with others	Comments about being able to do “normal” social activities: enjoying and trying to shake hands, playing with and picking up children
	Acclimation	Comments suggesting that participants were becoming more comfortable with having sensation, acclimating to the artificial sensation, learning to recognize and use sensory information, or suggesting a process of the sensory experience becoming more natural
	Reduced focus and attention	Comments about the degree of focus or attention required to complete tasks, or using the prosthesis more spontaneously with less thought
	Phantom position	Comments about the phantom sensation, including position of the phantom limb, changes in phantom position, or telescoping experience
**Self-Efficacy**	Confidence in tasks	Comments about participants’ confidence in their abilities and using the prosthesis to do tasks
	Usefulness of sensory information	Comments about how participants utilized the sensory information provided by the stimulation to do specific tasks
	Lack of confidence/ Distrust of the system	Statements in which participants expressed worry, uneasiness, concern, annoyance, or frustration with certain aspects of the system
	Control interference	Statements about inadvertent movement of the prosthesis or an inability to move, either due to stimulation interfering with myoelectric control or due to perceived stimulation interference
**Sensation Experience**	Sensation modality	Comments about the type, texture, or modality of the sensations provided by stimulation
	Sensation location	Comments regarding the locations of the sensations provided by stimulation
	Sensation timing	Comments about the perceived timing of the sensory percepts
	Sensation intensity	Comments about the intensity or magnitude of the sensations created by the stimulation
**User Attitudes**	Outcome acceptance	Statements about the participants’ desire to have the experience of a sensorized prosthesis rather than having no sensation, general statements about participants’ attitudes about the sensation or system, and statements about how sensation impacted their willingness to wear the system or their wear-time
**Stability of Sensation**	Changes in intensity	Comments about how intensity changed throughout the day or the study
	Changes in modality	Comments about the way in which the modality changed throughout the day or the study
	Changes in location	Comments regarding changes in locations of sensory percepts throughout the day or the study
	Changes related to arm position	Comments about how the sensation changed when the arm was in different positions
	Consistency of sensation	Comments about the general stability of the sensation, or that the sensation felt inconsistent or erratic
**System Operations**	Mechanical hand interactions	Comments about the physical properties and characteristics of the prosthetic hand and silicone sensors
	Stimulator program	Comments about the stimulation software program. These comments could refer to unexpected shutdowns or reboots of the stimulator, triggering of sensations when inappropriate, features of the program that the participants would like to see added or removed in future versions, or other issues that were caused by the stimulator program
	Connectors and cables	Comments about the ease of use, problems, or experiences referring to the cables and connectors in the system. Comments include issues related to cable snagging or tethering, connectors being pulled apart accidentally, and the experiences of the participants connecting and disconnecting the cables and connectors. Comments also include getting used to system donning and doffing procedures involving the cables and connectors
	Changing stimulation parameters	Comments about the experience of changing stimulation parameters, such as the pulse width and pulse amplitude. Comments about the frequency of changing parameters, ease of changing parameters, and rationale for changing parameters

The instances of overlap among the categories were calculated using NVivo. NVivo does this by finding segments of text that are coded into multiple categories. These text segments are then scored as an instance of overlap among the categories of which it is a member. The relationships uncovered in the coding process were discussed and used in the process of axial coding to identify the key relationships between major categories and subcategories [33]. Directionality of the relationships between categories was determined by examining the data contained in the overlap between pairs of categories. Axial coding was then discussed with the 4^th^ investigator (DT) who participated in the process of selective coding to define the central category that we believed represented the primary theme of the research [33]. The selective coding led to the development of a theoretical framework we named, “the impacts of sensation.” The key components of the theory were refined by re-reviewing the raw data for consistency and gaps [35]. In general, the categories excluded from the model were those that either did not interact with other categories or those that focused on the specific details and implementation of this particular sensory restoration system, and thus did not seem to reflect the broader concept of having sensory feedback.

## Results

Each category included in the theoretical model is described below, with examples from the coded text to illustrate the main concepts contained in each category.

### Return to normalcy

Comments pertaining to the overall category of return to normalcy were coded in the following sub-categories: naturalness of the sensation experience, focus and visual attention required when using a prosthesis, acclimation to the sensory stimulation, sense of embodiment of the prosthesis, phantom position, and using the prosthesis in interpersonal interactions. Except for the category of phantom position, which is described in S1 Appendix, each sub-category is described below.

#### Naturalness of sensation experience

Both participants felt that using the prosthesis with sensory stimulation made the experience of using the prosthesis feel “more natural” when compared to the prosthesis without sensation, and that this enabled them to do activities with less concentration.

*“I didn’t necessarily think about what I was grabbing and touching and everything*. *I’d think about the motion and my fingers moving and I could see them move and grab and feel when I touched*. *When the bend (aperture) sensor kicks in*, *it does have that tightening*, *so feels like the fingers*. *So I could visualize a lot better with having the sensation*.” *(P2)*

Remarks indicated that sensory stimulation was experienced as a normal way to receive tactile information, suggesting that sensory stimulation successfully replaced, at least in part, the sense of touch that had been lost. When asked about the sensory stimulation system, participant 1 said:

*“It was less of a system*, *and more of a normal function*. *If that makes sense*.” *(P1)*

With sensory stimulation, participant 2 felt that the experience of using his prosthesis was comparable to the experience of using an intact hand to do tasks. He said:

*“I just used the [prosthetic] hand*. *I just used the hand like I do this [intact] one*. *I don’t think about it*. *When I go to grab something*, *I don’t necessarily… I don’t necessarily think about it*, *I just reach and grab (picks up prosthetic sock with prosthetic hand) and keep going (puts down sock)*.*” (P2)*

The participants’ comments demonstrated the integration of the sensory stimulation information into their decision making processes, indicating that the tactile information was utilized in a comparable way to natural touch in their intact hand. This information enabled them to perform activities that they otherwise may have avoided due to concerns about squeezing too hard or inadvertently hurting others. For example, Participant 2 said:

*“Yeah*, *the two year old*, *she come up to me (gestures as if preparing both hands to pick up a small child) to be able to grab her and to be able to feel when I squeezed just a little bit to feel*. *Ok*, *I’m not squeezing too tight to pick her up (gestures with both arms as if lifting child)*, *and then bring her up this way (gestures toward left shoulder)*.*” (P2)*

Even though they had experienced sensory stimulation in the laboratory setting, participants commented that the experience of sensation became more natural when they used it daily at home.

*“No*, *it was*, *uh*, *I guess it seemed more natural… And not like it is here (gestures with both hands toward lab system)*. *It seemed like once I left here it was totally different*. *It wasn’t in a setting*, *controlled setting*. *I was able to do*, *I was able to grab***…***” (P1)*

#### Embodiment

Both participants remarked that when sensory stimulation was “on,” it made the sensorized hand feel “more real” and that they “could feel the object between (their) fingers.” Participants frequently referred to the sensorized prosthesis as “my hand” or “my fingers,” indicating their view that the prosthesis was a part of their own body.

*“… And it felt like it was my hand on the end of there (referring to the prosthetic socket)*.*”(P2)*

This experience of embodiment of the prosthesis was sustained even when the sensors were not constantly activated by touch.

*“Right*, *to me the hand was still there (gestures toward prosthetic hand using left hand)*. *Even with the stim off*. *Knowing that even if I just barely touch it (lightly touches right middle finger with left index)…*, *it would come on*. *My finger*, *my hand would be there*. *My fingers would be there*…*” (P1)*

The experience of embodiment was evident, even though the sensory percepts of tingling or pressure may have felt different than the intact hand.

*“Even though it was still tingling*, *it was me using my hand*, *you know*, *so to speak*.*” (P1)*

The experience of prosthetic embodiment appeared to be strongly associated with the sensory restoration. During the first portion of the home study where participants wore the device without sensation, participant 1 commented that it was “frustrating” and “feels like a tool.” During the last phase of the study when the sensation was turned off again, participant 1 commented that the prosthesis “does not feel like me–went back to being an attachment.”

#### Interaction with others

Both participants described how they utilized the prosthesis to shake hands with family, friends, or community members (both participants lost their right hands).

*“A couple of the teachers*, *professors*, *know what’s going on and they were curious about it*. *So we’re shaking hands left and righ*t.” *(P1)*

*“I shook a few people’s hands*. *One of the bankers that I’ve dealt with for 20 years*, *he come out and seen me there and he wanted to see the new hand*. *And I shook his hand*.” *(P2)*

Interacting with others using the prosthesis appeared to be a positive experience overall. Participant 1 said, “it felt good shaking other people’s hands.” Participant 2 commented:

*“It felt good to play with my grandkids and feel when they grabbed my hand and when I picked them up*.” *(P2)*

Participant 2 also described a few instances in which the sensory stimulation interfered with his myoelectric control, which prevented him from “let(ing) go” of someone’s hand while shaking it.

*“I mean*, *I shook the banker’s hand*, *and umm*, *ok*, *hold on*, *just a second*, *I can’t*, *because it wouldn’t let me open up*. *So I relaxed my arm for a second then it let me open*. *“(P2)*

Despite these occasional control issues, participant 2 stated that having sensory feedback made him feel “more confident” in using his prosthesis to shake hands, that he felt “more confident…than I have in years” and that he shook people’s hands “more often” when he had sensation.

*“Yeah*, *I felt more confident*. *I was shaking people’s hands more often and more confident about doing that and all*.*” (P2)*

Both participants described family and friends’ skepticism about the sensory stimulation system, which influenced the way that they interacted with others when sensation was enabled. For example, participant 2 described a “game” that his daughter devised to determine if he could actually feel the sensation as he claimed:

*“Oh*, *she*, *she’s like*, *how well can you really tell dad*? *Do those sensors really work*? *So I went like this (squeezes on finger sensors of prosthetic individually) so I knew which one*. *Yep*, *that feels like thumb*, *yep*, *that does feel like index*, *and what sensation do I got from that*. *I go*, *alright*, *you wanna know*? *Here (holds right arm behind back and looks away)*. *You touch ‘em and I’ll tell you which one you’re touching and she figured out that every time [she] touched*, *I could tell that you’re touching and I could tell that*, *which one she was touching*.*” (P2)*

#### Acclimation

Participant comments described an adjustment process in which they were getting used to having sensation, “getting more comfortable” or “in tune” with it, and learning to recognize and use sensory information in a more natural way.

Participants spoke about how they adapted to sensation, explaining that receipt of sensory stimulation for longer durations (as part of the take home study) altered the nature of their experiences, making the prosthesis “more intuitive” and “easier to use” or altering their interpretations of the sensory information. Participant 1 explained that the experience of using the sensorized hand and the sensation itself sometimes seemed “overwhelming” at first, but became more natural with home use. As he used the system, he reported that he “got used to it” and felt the sensory percepts more intensely. Because the feelings were “a little too much,” he had to “keep turning it [intensity] down.” After turning down the intensity, he began to feel that the sensation “was easy to use.”

Participant 2 described a similar process, where longer and repeated practice with the sensory stimulation made it feel more natural:

*“… the more I used it*, *the more natural it felt to me*.*” (P2)*

As he adapted, this participant described how he learned how to interpret the sensory percepts and began incorporating this sensory information when grasping objects.

*“And it did feel like contact touch and I’d feel mine’s adapting where it’s like*, *ok*, *that’s contact*, *so that’s where I want to stop at*, *don’t grab too tight*. *Grab a little tighter and I’d feel it a little* stronger.*” (P2)*

*“… But a lot of times it was just*, *(picks up bottle with left hand without looking at bottle) ok*, *I can feel*, *or yep*, *I can feel it so (puts down bottle) that’s where I’m going to stop at*. *If I start to pick up (picks up bottle with left hand while looking at bottle) and it’s loose (lets bottle slide downward through grasp)*, *ok*, *I’ll tighten down a little more (places bottle back on table)*.” *(P2)*

#### Reduced focus and attention

The participants stated that having sensory feedback decreased the amount of focus and/or visual attention required when using the prosthesis, which made grasping objects quicker, seemingly allowing them to perform activities with fewer interruptions. Participant 1 spoke about how he did not need to “look to see” what he was grabbing, instead relying on the sensory stimulation:

*“It was more natural*. *I didn’t even*, *I didn’t look to see (pretends to grasp coffee cup with prosthetic) where I was grabbing stuff*, *I just grabbed it … I had more control of it with less thought*. *If that makes sense*. *Cause I’d just grab and go*. *Instead of having to think about grabbing that (pretends to grab coffee cup with left hand) and going (gestures with left hand)*. *Because I knew the sensors were there*, *I knew the fingers would come on*. *I grab it and go (gestures with left hand*).*” (P1)*

Both participants contrasted the degree of focus they needed with and without sensation enabled. After using the system with sensation enabled, Participant 1 commented on how much focus he previously needed to exert when he did not have sensation. He said, “I always had to focus on what I was grabbing.” Participant 2 said:

*“Now [without sensation]*, *I have to watch to make sure that I’m over it*, *or how tight I’m squeezing*. *But with the sensors on*, *once I felt that I’s touching it*, *that’s where I’d stop at*.” *(P2)*

### Self-efficacy

The category of self-efficacy included comments pertaining to confidence (or lack of confidence) in using the prosthesis to do tasks. We identified the following sub-categories of self-efficacy: perceptions of the usefulness of the sensory information provided by the system, confidence (or lack of confidence) in the system and their perceived abilities, and control interference with prosthesis use. The sub-categories of “usefulness of sensory information” and “confidence in tasks” are included in our overall model and described below; the other sub-categories are described in S1 Appendix.

#### Confidence using the prosthesis to do tasks

Both participants indicated that having sensory feedback made them feel “more confident” in their abilities to use their prostheses to perform tasks. With sensation, participant 1, a right amputee, stated that he “had more confidence in doing things right handed” and that the system “seemed to give me more confidence in my right hand and less use of my left hand.” Even though the participants used the same prosthetic socket and controls with and without sensation enabled, participant 1 felt that the addition of sensory feedback improved his ability to control his prosthesis.

*“Felt like I was getting my sense of touch back and I was able to have better control over the prostheti*c.” *(P1)*

The sensory feedback appeared to affect confidence to do tasks, especially when the tasks involved handling or manipulating delicate objects. Participant 2 described carrying a coffee cup with the prosthetic hand while simultaneously doing another task with the contralateral hand:

*“Yeah*, *I felt more confident…More confident about*, *go to speedway and get a Cappuccino and carry it out (gestures with prosthetic hand as if trying to grasp something) with the prosthetic and use the other hand same time to open the door and do those sorts of things (gestures opening door with left hand*).*” (P2)*

Having sensory feedback impacted participant 2’s confidence in using the prosthesis to interact with others. During the sensation on portion of the study, he “was willing to try to shake people’s hands,” and he “felt more confident in shaking friends’ hands.”

#### Usefulness of sensory information

The information provided by the pressure sensors on the thumb, index, and middle fingers helped participants judge the timing of grasps with their prostheses. They used the pressure information to decide when to stop contracting their muscles and thus knew when to stop driving the terminal device to close, providing “better control over the prosthetic.” Participant 2 felt that the pressure information was useful while shaking hands:

*“I shook his hand (extends right arm as if reaching for a hand shake*, *then motions handshake) and it felt like*, *when it (the prosthetic hand) grabbed his hand that’s where I stopped at and that’s when I felt it*.*” (P2)*

Participant 2 also commented on using the pressure information to decide when he had gripped an object with a sufficient amount of force to pick it up:

*“…But a lot of times it was just*, *(picks up bottle without looking at bottle) ok*, *I can feel …that’s where I’m going to stop at*. *If I start to pick up and it’s loose (lets bottle slide downward through grasp)*, *ok*, *I’ll tighten down a little more*.*” (P2)*

The pressure information helped participants determine “how hard” they were “squeezing.” Participant 1 explained that pressure information was useful to him when he was grabbing delicate objects, enabling him to use appropriate force without harming the object. He said:

*“So it was neat using that to you know*, *grabbing my laptop*, *holding the lid up*, *not (pinches left hand fingers together)*, *because it’s a touch screen*, *you could tell when you squeeze hard on a screen you get that little indent*, *I didn’t see that*, *I stopped right there*.*” (P1)*

### Sensation experience

We coded comments pertaining to the experience of specific dimensions of the sensory percepts using the following sub-categories: modality, location, intensity, and timing. The sub-category of modality, part of our overall model, is described below; the remaining sub-categories are described in S1 Appendix.

#### Modality

Comments about the type or modality of the sensation were coded in this sub-category. Participants described the modalities of the sensations they experienced from the stimulation using the following descriptors: “tingling,” “pressure,” “vibration,” “contact touch,” “contracting,” and “tightening.” Participant 2 commented:

*“[The sensation is] beginning to feel pretty normal*, *feels like pressure*, *some contact*, *still has vibration*. *When I close [the hand]*, *those fingers are what is touching*.*” (P2)*

Participants explained how they often felt several simultaneous sensation modalities, though each modality component may have had a varying relative contribution to the overall sensation experience.

*“But when I had these fingers (points to prosthetic fingers using left hand)*, *and going it just felt like (moves left hand in pinch grip back and forth a few times) it was two sensations at once so to speak*: *the pressure and the tinglin*g.” *(P1)*

*“And it’s like light touch*, *or like (presses right thumb on table) if you’re resting… it felt like uh*, *if I had the intensity at like 3 or 4*, *the contact touch was at least a 2 or 3*. *That’s what I ended up noticing*. *It’s like*, *wait a minute*, *it doesn’t feel like it’s on the surface*. *It feels like I’m pressing down (presses onto table with right thumb) and feeling that* … *pressure underneat*h.” *(P1)*

Some modalities were perceived as more desirable than others. The tingling sensation was viewed as less desirable, though still better than having no sensation at all, as discussed in “outcome acceptance” below. Participants indicated that they would like to see changes to the sensation modality in future systems. When asked about what aspects of the system he would like to see changed, participant 1 said:

*“Probably the type of sensation… I think that would be the only thing*, *if I could change sensation*, *change the type of sensation*. *But that’s asking*, *asking for my cake and asking for another piece*.” *(P1)*

Participants 1 and 2 had differing experiences with the stimulation-evoked perception of prosthetic position. For participant 1, prosthetic aperture information was encoded as a tingling tactile sensation on his thenar eminence. For participant 2, aperture information was encoded as a kinesthetic sensation of hand closing. Participant 1 spoke about how the “annoying” tingling sensation modality contributed to his dislike of the sensation in his thenar eminence. Despite this annoyance he also explained that the aperture information was useful to him:

*“I think*, *you know*, *it seemed like… I don’t know if it just seemed more sensitive in that spot (gestures to left palm using right hand to point) and that’s why it was a little annoying*. *I think if I could*, *I would like to try to change the sensation to something other than that tingling*. *Maybe just a pulsing without the tingling component* … *Yeah I would still rather have that sensation*, *or that*, *information (points to thenar eminence using middle finger)*. *But maybe the type of sensation a little bit different*.” *(P1)*

In contrast, the aperture information for Participant 2 was mapped to a stimulation channel that evoked a kinesthetic percept of the hand closing. Participant 2 stated that this kinesthetic sensation felt “like it contracts” or was “tightening” such that it “feels like the fingers….are doing the movement.” He explained this modality of sensation was useful to him and helped him appropriately interpret the information from the prosthetic aperture sensor.

*“With the sensation though… the bend (aperture) sensor*, *because it feels like it contracts*, *feels like it’s (the hand has) reached in and grabbed around things*.” *(P2)*

### User attitudes

Comments coded in the “user attitudes” category pertained to the participants’ views of the benefits of sensory stimulation (or lack of benefit) and the impact of the sensory stimulation system on prosthesis wear time.

#### Outcome acceptance

Participants expressed clear preferences for having sensory stimulation and talked about how having sensation positively impacted their desires to wear their prostheses. Participant 1 stated that the sensation experience was “better than I thought it was going to be” and shifted his emotional outlook to a “better feeling overall.” Participant 2 said, “I enjoy that sensation of having, being able to feel my hand,” and commented:

*“When sensation is on*, *I can feel my hand*, *I can feel the objects I am touching*. *It felt better doing the tasks with sensation on*.*” (P2)*

Both participants commented that it “felt good” to have sensation and that the sensory feedback improved their overall experience with the prosthesis. Participant 2 described how the tactile sensation in his fingertips contributed to his positive experience with the system.

*“With the sensation though*, *you know (picks up sensor calibration block with left hand)*, *being able to feel (places foam block in prosthetic hand grasp) that I’ve grabbed it and I’m holding i*t… *And then feeling it in the fingertips*, *and everything*, *makes a big difference*.*” (P2)*

Both participants commented that they wore their prostheses for longer periods of time because they liked having the sensory stimulation. Participant 2 said, “I’ve been more apt to wear it and more willing to wear it, being able to feel that sensation.” Participant 2 also reported that he often wore the prosthesis for most of the day because he enjoyed having the sensation, and that he only doffed the system when his socket became bothersome:

*“That’s because I like the sensation of having my hand there, and feeling like my hand was there*, *so I didn’t want to take it off (laughs). There’s a few days I took it off (gestures as if removing socket) after so long just because (presses on residual muscles of forearm) the socket was bothering me. It wasn’t because I wanted to take it off, because I didn’t want to, but eh, the socket’s bothering me enough, I need to take it off today (gestures as if pulling off socket) …” (P2)*

Both participants commented that they used their prostheses more often to grasp objects just so that they could feel the sensation. Participant 2 explained how he found that he wanted to “grab a little more…because I want to feel the sensation.” He reported that he repeatedly touched the fingertips of the sensorized hand, both to confirm whether the sensors were in functioning order and to feel the sensation.

*“… I had a tendency during the day if I held it closed for a while and it shut off*, *and I’d be sitting there for a while. Ok, let’s see, (gestures as if squeezing on fingertip of missing hand) yeah, that one feels good (…) Feel it to make sure they’re still working and just to feel it.” (P2)*

Both participants were very positive about the provided sensation, even though they occasionally commented that they might have preferred a different sensory modality (such as pressure over tingling). Although the sensory modality was not always ideal, both were clear that having a tingling sensation was “better than nothing.”

*“No*, *it’s better than nothing … I like the pressure component of it, toward the end of the week. I would like to see that, if possible. But if, I mean, it’s still a prototype… But, over nothing, tingling is fine….” (P1)*

Participant 2 also remarked that even though his prosthetic socket fit loosely and that his prosthesis operated in the same manner with and without sensation, it felt better with sensation:

*“Even though it’s still got that wobble (shakes residual limb as if socket were loose on arm) to it and a little cumbersome on certain things it still felt better than no sensation at all and not feeling like a hand was there*.*” (P2)*

When the home study was ending, Participant 2 lamented about the looming loss of the sensation stimulation. He compared the loss of sensation stimulation at home to losing his hand again:

*“… tonight’s my last night with it*, *when I come back, I gotta wear it another 3 weeks, but that’ll be without sensation. That’s like losing your hand all over again!” (P2)*

### The impacts of sensation on the experience of a hand

**Fig 2:**
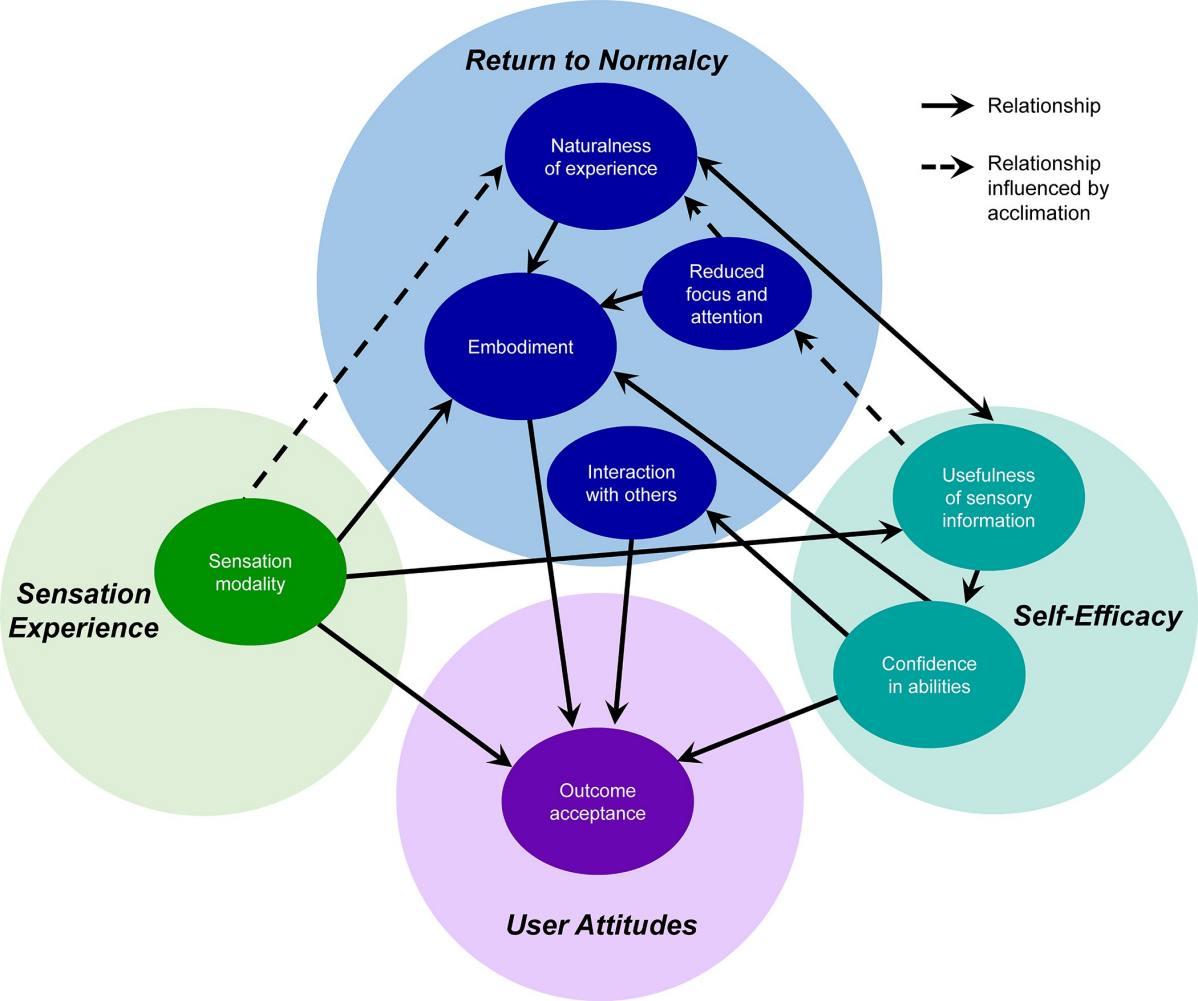
Model of the benefits of sensation on the experience of a hand. Concept categories are indicated by color. Solid arrows indicate directional relationships between concepts. Dashed arrows indicate relationships that are influenced by acclimation and thus strengthen over time.

We labeled the core category generated from the analysis, “*the impacts of sensation*.” This category characterizes the lived experience of having a sensing, embodied, prehensile upper limb, rather than an insensate prosthetic tool. Our resulting theoretical model explains the major factors contributing to this complex process. The full model (Fig 2) incorporates key sub-categories of ***return to normalcy*, *self-efficacy*, *sensation experience*, *and user attitudes*.** The critical categories of ***return to normalcy*** included in the model are the perceived *naturalness* of the sensory experience, the experience of *embodiment* of the prosthesis, the *focus and attention* required to use the prosthesis, and the use of the prosthesis in *social interactions*. The model shows the relationships between these categories to two key aspects of ***self-efficacy*,** which we call *usefulness of sensory information* and *confidence in abilities*, and an aspect of ***user attitudes*,** which we call *outcome acceptance*. The influence of *sensation modality*, a key aspect of ***sensation experience***, on these relationships is also shown. The model demonstrates the directional relationships of these categories, as determined by analysis of the text contained in the overlap between categories. The sub-category of *acclimation*, part of ***return to normalcy***, is a process that strengthens or modulates the relationships between some categories over time. There are four main nodes in our model, each of which interacted strongly with several other categories. These four nodes are: *naturalness*, *usefulness*, *embodiment*, and *outcome acceptance*, and each are described in more detail below.

**Fig 3:**
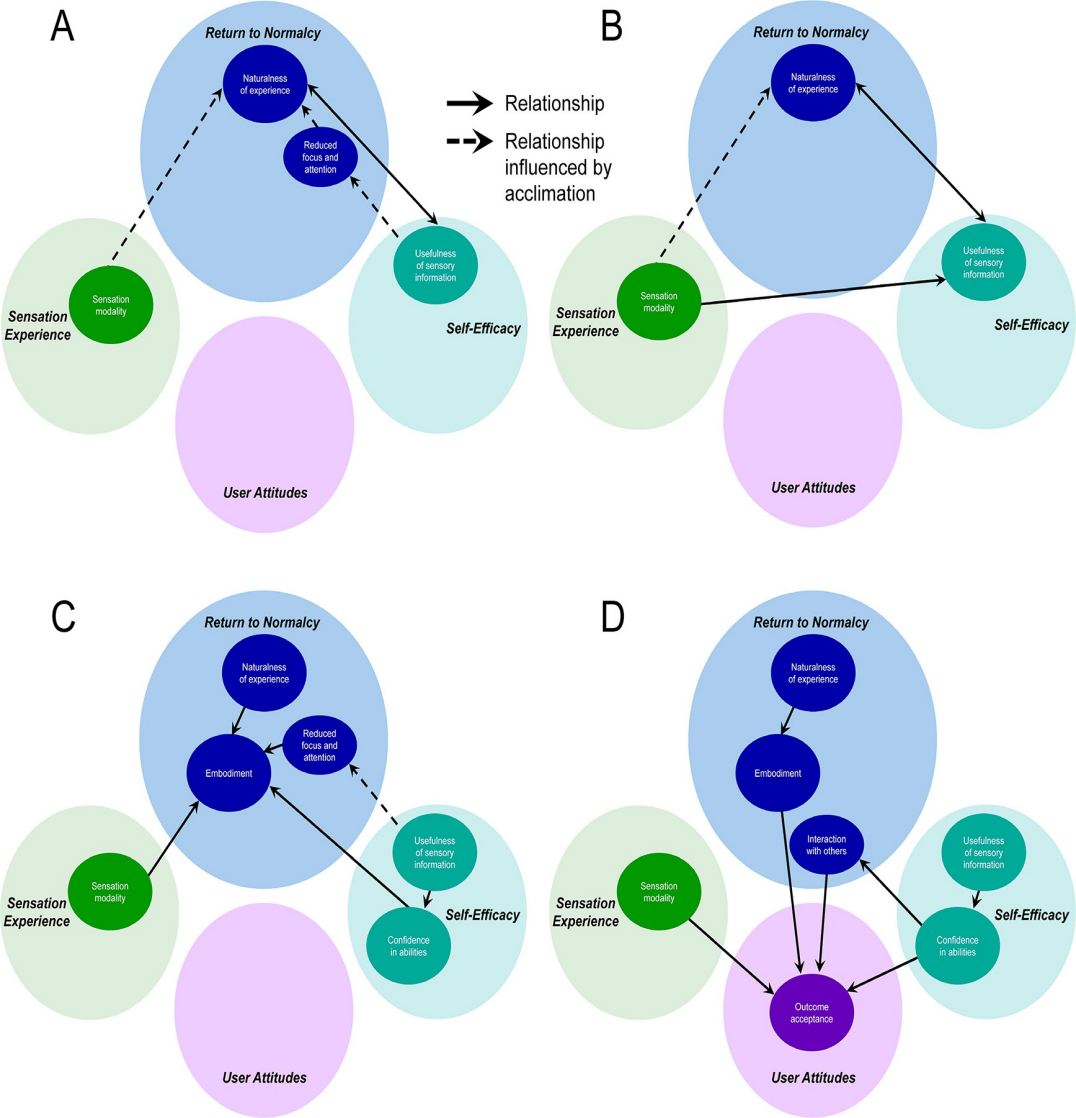
Concept relationships governing the benefits of sensation on the experience of a hand. A) Determinants of naturalness of experience. B) Determinants of usefulness of sensory information. C) Determinants of embodiment. D) Determinants of outcome acceptance. Solid arrows indicate directional relationships between concepts. Dashed arrows indicate relationships that are influenced by acclimation and thus strengthen over time.

Perceived *naturalness* largely results from the experience of *sensation modality* provided by the SRS and the *usefulness of sensory information* (Fig 3A). The sensory percepts become increasingly more natural feeling through the process of *acclimation*, which is achieved as the user garners experience using the system in everyday life. The *usefulness of sensory information* enables more spontaneous and fluid prosthesis use, attained, in part, as the user returns to a more normal state, with a greater sense of naturalness facilitated by reduced dependence on visual information when using the prosthesis (i.e. the category of *reduced focus and attention*) and a lessening of cognitive burden, both of which are also enhanced with *acclimation*. Thus, everyday activity performance with the SRS becomes less mentally taxing, and the experience of the sensorized prosthetic hand becomes more natural.

The perceived *naturalness* of sensory experience also contributes directly to increasing the perceived *usefulness of the sensory information*, because information from more natural feeling percepts is more easily utilized (Fig 3B). Certain sensory *modalities* are particularly valuable in enhancing the usefulness of the supplied sensory information; sensory information is more useful when it is conveyed via a modality that matches the physical nature of the information (for example, prosthetic aperture information is more useful when the associated evoked sensation feels like joint movements rather than tingling or some other tactile modality).

Prosthesis *embodiment*, or the experience that the prosthesis is a part of oneself, is created directly through the perceived *naturalness* of the sensory experience, through the *modality* of the sensory percepts, and through a greater sense of *confidence in abilities* (Fig 3C). *Confidence* is enabled by the *usefulness of sensory information* in everyday life. The *naturalness* of the experience of having a hand that can feel transforms the prosthesis from an extracorporeal tool into a normal, animate hand. Having sensory information that was *useful* in performing tasks yields increased *confidence* in their abilities to do tasks with the prosthesis. Higher *confidence* supports the perspective that the prosthesis is a capable and rightful part of the body. The *reduced focus and visual attention* required to operate the prosthesis, facilitated by the *usefulness of the sensory information*, further strengthens the perception that the prosthesis is a natural part of the body.

The model culminates in the category of *outcome acceptance*, which is characterized by satisfaction with the SRS and a desire to have and use it (Fig 3D). *Outcome acceptance* is driven by one’s *confidence in abilities*, the sense of prosthesis *embodiment*, the overall gratification created from restoration of hand *sensation modalities*, and enhanced *interaction with others*. The *usefulness of sensory information* indirectly leads to *outcome acceptance*, since it can enable greater *confidence in abilities*, which also impacts one’s *interactions with others*. The sensation experience, and in particular *sensation modalities* (even those considered less than ideal), was integral to the participants’ attitudes and *acceptance* of their situations. The *naturalness* of the experience of having a hand that can feel, through its contribution to embodiment, also contributes to *outcome acceptance*.

## Discussion

### Understanding the perceived benefits of sensory restoration

Our analysis produced a theoretical model that describes the perceived benefits of sensation on the overall experience of having a hand. This is the first study to examine the personal experiences, opinions, and attitudes of upper limb prosthesis users who used prosthetic hands with multi-location, naturalistic, somatotopically-matched sensory feedback. We believe our model will be useful for understanding the impact of restoring sensation to upper limb amputees, especially through peripheral nerve interfaces.

Major components of our model are supported by the quantitative data collected during the take home study and presented in a parallel publication [22]. During the home study, participants completed daily surveys about their experiences and sensations. In laboratory testing sessions, they completed additional surveys related to perceptions of prosthesis use and performed standardized functional tasks with the prosthesis. Surveys included the Orthotics Prosthetics Users’ Survey (OPUS) Upper Extremity Functional Status (UEFS), which measures the perceived difficulty of doing activities of daily living with the prosthesis; the Patient Experience Measure (PEM), which measures perceptions of self-efficacy (confidence in abilities), prosthesis embodiment, social interaction, body image, and prosthesis efficiency; OPUS Quality of Life (QoL) survey, which measures quality of life; and the Quick Disabilities of the Arm, Shoulder, and Hand (QuickDASH), which measures perceived disability. Throughout the study, on-board data logs recorded wear time and prosthesis sensor activity [22].

Survey data from the PEM and OPUS UEFS demonstrated that after using the sensory restoration system at home for a week, participants had significant improvements in self-efficacy, prosthesis embodiment, and perceptions of their abilities to use the prosthesis in social interactions. This supports the key impacts of sensation, elucidated in the theoretical model, on the categories of confidence in abilities, embodiment, and interaction with others. Prosthesis sensor data indicated that participants wore their prostheses longer and used their prostheses more frequently to grasp or interact with objects when sensation was enabled, substantiating the participants’ statements about the impact of sensation on wear time. Finally, participants’ perceived disability scores on the QuickDASH were significantly decreased and quality of life scores on the OPUS QoL were significantly increased after home use with sensation, which quantitatively demonstrate the impact of sensation on aspects of outcome acceptance [22].

We believe that our theoretical model helps frame and interpret findings from prior studies of sensory restoration. Our model can also be used to facilitate the synthesis of results across previous and future sensory restoration studies by grouping common concepts and describing interactions among experimental variables. The model may be applied to identify themes from larger data sets that may reflect higher-order changes in perceptual or cognitive processes due to sensory restoration. Additionally, the model provides a framework that can be used to inform the development of novel instruments that measure important constructs related to sensory restoration.

Our study also uncovered participant views on which technological aspects of the sensory feedback system need to be improved in future design iterations. Robustness was a concern for both participants, and both avoided using the device to do outdoor activities that may have damaged the system (see “Lack of confidence/distrust of system” in S1 Appendix). Participants experienced instances where the sensory stimulation interfered with their myoelectric control (see “Control interference” in S1 Appendix), suggesting that future engineering efforts should address strategies to reduce stimulation artifact in EMG recordings. There were also several issues that participants identified with the software of the stimulator and the cabling of the system components (see “System Operations” category in S1 Appendix). For example, the participants were able to independently calibrate their stimulation levels as desired each day, but did not like that the levels reset to predefined settings when the stimulator was power cycled. Researchers and engineers should incorporate this user feedback into their designs for future sensory restoration systems in order to improve the user experience, which may also influence outcomes.

### Comparison to upper limb amputee outcome acceptance

In keeping with grounded theory methodology, we were largely naïve to the qualitative literature on models of outcome acceptance in upper limb amputees during our data analyses. After completing our analyses, we conducted a post-hoc literature search on the topic. We found remarkable similarities between our model and other models presented in the literature on upper limb prosthesis use.

For example, key components of our model of outcome acceptance mirrored the major themes identified in Murray’s phenomenological study of successful prosthesis users, despite the fact that our participants had sensory restoration whereas those in Murray’s study did not [27]. Our sub-categories of “acclimation” and “focus and visual attention” parallel the themes “adjusting to a prosthetic” and “awareness of the prosthesis,” respectively. In “adjusting to a prosthetic,” Murray discussed the process through which a user becomes familiar with the prosthesis, both physically and perceptually. In “awareness of the prosthesis,” Murray described how users experienced a gradual decrease in the amount of attention and concentration required in prosthesis use, and an increasing “naturalness” of prosthesis use with experience. Similarly, we saw a direct relationship between “focus and visual attention” and “naturalness of experience.”

Comparison of our model to themes identified in prior studies of new prosthesis technologies also revealed several parallels. A study by Luchetti and colleagues examined outcome acceptance for persons who used a Michelangelo prosthetic hand for six months [26]. In Luchetti’s study, the participants described their myoelectric device as “essential” to their lives, and expressed feeling useful with the device donned [26]. Our participants also expressed a similar sentiment of wanting to wear the sensorized prosthesis for longer periods and to use it more frequently to grab objects, which contributed to our theme of “outcome acceptance.” As in our study, Luchetti et al. found that the use of a prosthesis to engage in social interactions, such as shaking someone’s hand, was important to participants and linked to outcome acceptance. However, the motivation of our subjects for engaging in social interactions with the sensorized prosthesis was driven by the prosthesis feeling natural (naturalness of experience) and like their hand (embodiment) due to the sensory restoration. In contrast, Luchetti and colleagues surmised that the social interactions of their participants were encouraged by the “normal” appearance and functionality of the Michelangelo Hand [26].

A phenomenological study by Lundberg and colleagues examined the experience of living with an osseointegrated prosthesis [11]. Lundberg et al found that the enhanced functional capabilities enabled by their technology allowed participants to be “more engaged in their life and social interactions” [11], which aligns with our finding that sensation led to improved “social interactions.” Lundberg and colleagues reported that their participants stated that osseointegration allowed them to “not need to spend time and mental energy” on their prostheses, which the authors posited contributed to the functional changes enabled by osseointegration. This finding aligns well with our “reduced focus and attention” category. Just as our participants experienced “distrust of the system” in some cases (see S1 Appendix), the participants with osseointegration felt “caution” in regards to their devices and “fear of losing the improved function.” For both the sensory restoration and osseointegration studies, the participants were early adopters working with new prototypes and imperfect devices. These feelings of caution and distrust may reflect the participants’ reasonable acknowledgement of the risks they undertook while living with the intervention, or they may reflect the strong preference for having the new intervention compared to not having it due to malfunction or breakage.

Finally, Lundberg and colleagues posited that osseointegration enabled “a gradual change in their identity from considering oneself as being disabled to having an identity as a healthy person.” This concept is parallel to our domain of “return to normalcy,” and in particular the sub-categories of “acclimation” and “naturalness of the experience.” In our study, the participants’ comments demonstrated that they felt that sensation began to restore them to a more “normal” state, that is, the state of an able-bodied person. We divided this “normalcy” concept into several sub-categories, one of which was the “naturalness of the experience,” which described that the sensation itself felt “natural”, that the sensation arising from touch on the prosthetic hand seemed like a “normal” way to get information, and that the sensation enabled the overall experience of the hand to be more “normal.” We defined the category of “acclimation” as a gradual process that enabled improved naturalness of the experience over time. In both osseointegration and sensory restoration, perhaps the gradual rate of these changes is essential to the profundity of the impact of the intervention.

### Implications for embodiment

In the literature, discussions of embodiment encompass the related, but differentiable concepts of body ownership, body image, and body schema. Body ownership, also called self-attribution [36] or self-identification [37], is the conscious experience that the body belongs to oneself and the identification of one’s body as distinct and perceptually unique from other people’s bodies and external objects [37–39]. Body image refers to the conscious perception of one’s body, including perceptual experiences, beliefs, and attitudes about the body [40]. In contrast, the body schema is a preconscious internal model of the body, which consists of neural representations of motor functions and multimodal sensory inputs [40,41]. Incorporation of a device into the body schema involves changes to brain representations, changes in sensory processing, and inclusion of the device in preconscious sensorimotor models [41–45]. However, a conceptual distinction is often made between true bodily incorporation, in which an object becomes part of the body, and bodily extension, in which an object is an extension of the body. [42,46]. True incorporation of non-corporeal objects into the body is thought to only be possible where the object substitutes for a missing body part and thus contributes to body completion or wholeness, such as with prostheses for persons with limb loss [42]. Bodily extension, in contrast, occurs with most forms of skilled tool use. Although bodily extension also involves changes to sensorimotor models, it is temporary and depends upon the location of the object relative to the body [42,46–48].

Our category of embodiment primarily consisted of the concepts of body ownership [42] and changes in the conscious perception of the body image [40]. Participants’ comments referring to the prosthesis as “my hand” or “my fingers” demonstrate self-attribution and a sense of ownership. Participant statements that “it felt like my hand was there” or other descriptions of their hand being continuously present speak to a body image that now includes a hand. The participants frequently contrasted the experience of having a “hand,” when sensation was enabled, with having a “tool” or “attachment,” when sensation was disabled. This also reflects a perceptual shift towards body ownership and inclusion of the prosthesis into the body image when sensation is provided.

Participants did not directly discuss body schema in this study, which is expected since the body schema operates largely outside of conscious awareness [42]. However, indirect evidence suggests that the participants likely experienced true bodily incorporation of the prosthesis. Some of the participant experiences categorized in the “reduced focus and attention”, “naturalness of experience,” and “confidence in abilities” categories likely represent consciously perceivable consequences of changes in the body schema due to incorporation of the prosthesis [40]. With the stimulation-evoked sensory feedback included in sensorimotor models of hand interactions, participants were able to grab objects with less focus (see “Reduced focus and attention”). This speaks to the “transparency” in tool experience that occurs for incorporated devices, where the tool enables an experience but is not itself the focus of the experience [42,43]. As the participants gained experience with the system and the sensory feedback became more natural, the participants began viewing the prosthesis sensation as a “normal function,” and were able to incorporate it into their decision-making processes (see “Naturalness of experience”). Further, even though we did not modify the control scheme of the prosthesis in this study, participants claimed that they had “better control” of their prosthesis and that they had “more control with less thought” (see “Confidence in abilities”). These comments suggest that the sensation was able to close the loop in their internal sensorimotor control models, at least in part, reflecting the successful incorporation of the sensory-enabled prosthesis into the body schema.

### The critical role of embodiment

Our model demonstrates that embodiment is critical for outcome acceptance. The pivotal role of prosthesis embodiment in prosthetic outcome acceptance has been acknowledged by others who have written about the importance of limb ownership, body image, and prosthesis incorporation into the body [27,37,47]. Prior studies describe prosthesis users’ desire for a “corporeal” rather than an “extracorporeal structure” or “inert supplement” to the body. This speaks to prosthesis users’ preference for a device that becomes a part of one’s own body through bodily incorporation, rather than an extension of the body schema or a tool [49]. The importance of embodiment also emerged from prior qualitative and phenomenological studies of successful prosthesis users [27], users of osseointegrated prostheses [11], users of dexterous prostheses [26], and the experience of living with limb loss [29]. Body image has been noted to contribute to psychosocial adjustment to amputation, especially in women [25,50]. In addition, a Delphi study of rehabilitation professionals and upper limb amputees identified several factors related to body image and incorporation as indicators of successful prosthesis outcomes and important factors to consider in rehabilitation [51].

Our model shows a direct relationship between sensation modality and embodiment, supporting the assertion that a primary condition for prosthesis incorporation into the body is that the prosthesis becomes a “knowing body part” [42,43]. The “knowing” provided by the sensory stimulation in our study clearly contributed to the sense of embodiment. This is supported by prior studies which found that touch and proprioceptive signals are important for establishing body ownership in the rubber hand illusion [36,52–54]. The relationship between sensation and embodiment was demonstrated in a prior qualitative study of upper limb prosthesis users, which included those who had experienced vibrotactile or mechanotactile sensory substitution on the residual limb. These subjects reported that having sensation is important to establishing prosthesis incorporation [30]. Another qualitative study of the experience of osseointegrated prosthetic limbs focused on the varying degrees to which participants incorporated their prostheses into their sense of self, further highlighting the importance of prosthetic incorporation to amputees [11]. Sensation likely played a role in the incorporation of the prosthesis for these participants, given that participants reported improved proprioception, gained an ability to feel through the skin of the residual limb that was no longer encased in a socket [11], and likely experienced bone-conveyed sensation via osseoperception [12].

The relationship between sensation and embodiment has also been demonstrated in prior studies of amputees with targeted sensory reinnervation [55], sensory feedback provided through electrical stimulation [21,56,57], and proprioception provided through surgical coupling of agonist and antagonist muscles [58]. Interestingly, with targeted sensory reinnervation, proprioceptive sensation alone did not provide a sense of embodiment of a prosthesis [59], while cutaneous touch feedback was able to elicit a sense of embodiment [55]. Our subjects experienced both tactile and proprioceptive sensations during the home use study. The interaction of sensation modality with embodiment in our theoretical model may reflect that these modalities contributed to prosthesis embodiment to different extents.

Although our study and prior studies found that sensation played a critical role in prosthetic embodiment, sensory experience may not be an absolute requirement for embodiment. Murray described how some, but not all, successful prosthesis users experienced the prosthetic limb as a corporeal structure (part of themselves), even though these users did not have sensory feedback [27]. Luchetti and colleagues also observed the theme of embodiment among their participants who utilized a novel prosthetic hand without sensation, and attributed it to the hand’s natural appearance and enhanced dexterity [26]. Body congruence, i.e. that objects resemble body-parts, is considered by some to be a necessary element of successful object incorporation [36,60]. Training and learning with a prosthesis may also enable increased prosthesis incorporation into the body schema, even without sensation [61,62].

However, these disparate findings regarding the role of sensation in embodiment may be resolved by considering that there may be varying degrees of embodiment and that the likelihood of embodiment also differs across persons. De Vignemont describes a “manifold” of various types of embodiment, proposing that there are differing degrees of embodiment [47]. This idea of varying degrees of embodiment is supported by studies of anatomical congruence of embodied objects [60] and the conceptual distinction between true bodily incorporation and bodily extension described above [42]. In addition, not all prosthesis users currently experience embodiment [27]. While both participants in our study provided strong evidence that they experienced limb ownership and bodily incorporation of their prostheses, approximately half of the participants in a prior qualitative study of osseointegration did not experience full incorporation [11].Thus, although prosthesis functionality, appearance, and training may contribute to prosthetic embodiment, they are not always sufficient. The addition of sensory restoration to prostheses could lead to greater likelihood of prosthetic embodiment in upper limb amputees and enhance the degree of embodiment for those who experience it.

### Implications for phantom pain

Phantom limb pain is another aspect of the experience of limb loss that has been well-documented and may be a factor related to outcome acceptance [63]. Because our participants did not experience phantom limb pain after the start of the study [15], we could not study the impact of sensation on phantom limb pain or the interaction of phantom limb pain with other concepts in the model. However, based on evidence from prior studies of electrical stimulation of peripheral nerves, sensory restoration may be an effective treatment for phantom pain [15,64–66]. We hypothesize that daily exposure to sensory restoration through at-home usage of a sensory-enabled prosthesis will yield further reductions in phantom pain beyond those achievable in laboratory settings. Interestingly, limb telescoping, which is the experience of the phantom limb location retracting into the stump [67,68], was significantly decreased for our participants when sensation was enabled during the home study, such that the location of the phantom limb attained a more natural extended position when they were provided sensory feedback [22]. Limb telescoping is correlated with phantom limb pain, and although the neural mechanisms are not fully understood, both telescoping and phantom pain are related to cortical reorganization in the somatosensory cortex [68,69]. Sensory feedback may reverse the cortical reorganization of phantom limb areas, leading to decreased phantom pain and improved body representation [70]. In our home study, when sensory feedback was again disabled in Stage 3, the telescoping experience returned and the phantom limb retracted towards the stump [22]. This suggests that continued exposure to behaviorally-relevant sensory feedback may be necessary to maintain the cortical organization changes induced by sensation.

### Limitations

Our model was developed from data derived from the lived experiences of two male research participants with upper limb amputation who had a sensory restoration prosthesis system. Both research participants had the same neural interface for sensory restoration, the same sensory restoration system, and the same prosthetic hardware. Both had transradial amputation due to trauma. Therefore, we cannot tell to what extent our model will generalize to other sensory restoration systems, neural interfaces, terminal device types, to women amputees, or to other patient populations.

We acknowledge that the model may be incomplete and limited because of certain aspects of our data collection methodology. Data collected via free response survey questions may have been limited by the ability of the participants to write their answers with their non-dominant hand or prosthesis, given that both lost their dominant hand due to amputation. In addition, daily diary responses may have resulted in over-representation of certain concepts, since participants may have chosen to write about similar themes repeatedly across days. That said, the majority of the data we analyzed in this study was accrued through participant verbal responses to unstructured interviews. Thus, the model may overemphasize concepts uncovered through interview. The unstructured interviews were collected by several interviewers, each of whom had their own style of question phrasing and probed responses differently. It is possible that a more structured interview approach with standardized probing and questioning would have yielded further findings across participants. Additional research, using a larger patient population and a more structured approach, is recommended to explore and confirm this model.

Another limitation is that we did not collect any quantitative metrics of user attitudes or acceptance of the sensory-enabled prosthesis. The theoretical model that we present clearly suggests that surveys measuring user attitudes or prosthesis acceptance should be collected in future home use studies of sensory restoration prostheses. Our study only examined the impact of sensation on outcome acceptance. Thus, the interaction of sensation with other factors that have been previously identified to be important to outcome acceptance, such as hand dexterity and appearance [26], cannot be determined. Because our participants used the same single degree-of-freedom prosthesis with the same control scheme in this study, we cannot tell to what extent hand dexterity or control scheme impacted sensation experience or outcome acceptance.

Our model may also have been impacted by the perceived reactions of family and friends to the sensory restoration system. Family and social support has been shown to be critical for psychosocial adjustment to amputation [25,50,71,72]. Not surprisingly, given that this was the first study of a sensory restoration prosthesis, both participants described the skepticism of their family and friends in the functionality of the sensation. It is possible that the skepticism of the participants’ family and friends influenced their perspectives on sensory feedback, their views of the sensory-enabled prosthesis, and their choices in how to respond to survey and interview questions. Future studies of sensory restoration prostheses may yield different outcomes or demonstrate differences in our model once sensory restoration becomes more widely-known and accepted by the public.

### Application of the model

The experience of having a hand is a complex phenomenon. In this study, participants had an experiential shift in which their prosthesis moved closer to being perceived as a normal hand due to the addition of sensation. The theoretical model of transformation of hand experience may serve as a starting point for further improvements to rehabilitation following limb loss.

This model could guide researchers, clinicians, or engineers in determining how best to improve outcome acceptance using sensory restoration, other novel prosthesis designs, or therapeutic interventions. For example, from the perspective of an engineer, efforts could be made to enhance outcome acceptance by designing a new sensory encoding algorithm (to increase the “usefulness of sensory information”) and/or by making the user interface easier and more intuitive to use (to increase the “naturalness of the experience”). Such efforts can then be evaluated by examining end-users’ “confidence in abilities” or prosthesis “embodiment.” From the perspective of a rehabilitation therapist, efforts could be made to improve training on use of the sensory information (to increase the “usefulness of the sensory information”) and then studied by measuring the impact of such training on “confidence in abilities” and “interaction with others.”

The similarities between our model and prior models may reflect over-arching viewpoints and needs of the amputee community and suggest a common theoretical framework to describe outcome acceptance in upper limb prosthesis users more broadly. These findings support the potential transferability of our model to all upper limb prosthesis users, and its application to aid in understanding the impact of novel prosthesis technologies and rehabilitation interventions on outcome acceptance. We recommend that the relationships outlined in our model be explored and tested in other studies of upper limb amputees to determine if the model is applicable and useful.

Though this theoretical framework was developed to explain the experience of upper limb amputees, it may be applicable to individuals with various conditions involving sensory deficits of the hand. For example, following a stroke, nerve injury, or spinal cord injury, a person’s perception of their hand or limbs may be substantially altered. One qualitative study of the experience of persons with neonatal brachial plexus injury identified several key topics that overlap with domains in our model, such as sensation experience, arm/hand compensation and preference, explaining functionality/appearance to others, self-esteem, and body image [73]. This provides evidence that there are universal aspects of the experience of living without sensation in one’s hand that are independent of etiology. Our model may also be useful for understanding the experiences of persons who use other types of assistive devices, such as wheelchairs [74]. The experience of human-device integration, which we examine here through sensory-enabled hand prostheses, likely shares common themes across devices and patient populations.

## Supporting information

S1 Appendix Descriptions of sub-categories not included in the theoretical model. (DOCX)

S2 Appendix Survey questions and interview question examples. (DOCX)
