# Supplementary material for: The benefits of sensation on the experience of a hand: A qualitative case series
Source: PLoS One. 2019 Jan 31;14(1):e0211469. doi: 10.1371/journal.pone.0211469 (PMC6355013; doi:10.1371/journal.pone.0211469)
Supplement: S1 Appendix — (DOCX) [file pone.0211469.s001.docx]

**S1 Appendix.** Descriptions of sub-categories not included in the theoretical model.

RETURN TO NORMALCY

***Phantom Position***

After using the sensation at home, Participant 2 compared the position of his phantom hand to the position that a hand would be in if it were holding various types of sporting balls.

*“There’s a few days you’ll see I wrote down, the hand felt more open, it felt like it was open like this, like I was holding a football. The last couple days it’s been a little tense and tightened up like holding a golf ball. And sometimes the softball and baseball. But 2 or 3 different times it open up, it felt relaxed. I mean right now it feels pretty relaxed.” (P2)*

Participant 2 also commented on his experience of limb telescoping experience, describing where he perceived his phantom fingertips were located relative to his intact hand or relative to the prosthetic limb.

*“…I had been putting my phantom fingertips back here (gestures toward palm of left hand with residual); it felt more like the fingertips were out where they should be (gestures toward ends of fingertips of left hand). I could close my eyes and touch (moves left hand through air in front of end of residual stump) my fingertips, and not be back (gestures at end of stump with left hand) four or five inches.” (P2)*

Participant 2 commented that using the sensory restoration system seemed to change the degree of limb telescoping that he experienced. After using the system, he perceived that his phantom fingertips “were out where they should be,” rather than telescoped towards the residual limb several inches.

*“Yeah, it actually got to where it felt like the fingertips were out here (gestures to fingertips of prosthetic hand with left hand), not back here (gestures to MCP joints of prosthetic fingers).”*

SELF-EFFICACY

***Lack of confidence/distrust of the system***

Participant 1 explained that he initially was very wary about making changes to the system settings, saying he didn’t “want to mess” with it, because he didn’t want to “screw” it up. He worried that the sensory restoration system would inadvertently “make the hand open and close,” or “act up.” This lack of trust impacted the way that he used the sensorized hand. For example, when the interviewer asked him whether he used the sensorized hand to touch his wife, he explained that he did not because he feared the UECU, or “the box,” would misbehave:

*“… I didn’t do it, cause I think I was more worried about the box acting up more than anything else.” (P1)*

Concerns about inadvertent movement and the need to protect the prosthesis from damage mirrored concerns about their own prostheses. For example, participant 1 mentioned that the prosthetic terminal device would open inadvertently when he walked near an electrical outlet:

*“Yeah. It’s the hand. It’s actually.. my (own prosthetic) hand does the same thing . For some stupid reason, if I’m carrying something and I go near an outlet, it goes *phoo* (opens up left hand wide, looks down at ground as if dropped something).) We’re having scrambled eggs honey.” (P1)*

Participant 2 mentioned that he would not wear the device while doing yard work, and participant 1 mentioned that he did not use his prosthesis when working at his camper.

*“I mean, there would be certain days I wouldn’t because there are certain days I don’t even wear my prosthetic. If I’m outside. I don’t even wear it when I’m outside doing yardwork because a lot of times mowing the grass with a push mower…I just don’t want it out when I’m doing that because I don’t want the dirt and dust.” (P2)*

***Control interference***

The “Control Interference” sub-category contains participants’ comments about inadvertent prosthesis movement or inability to move the prosthesis due to stimulation interfering with myoelectric control. Participants talked about how they “calibrated” or “(re-) adjusted” the stimulation/ intensity, but felt that the sensation would, on occasion, “still be too strong,” and that this sometimes caused interference with the prosthesis function. Sometimes the stimulator became “frozen” and the prosthetic hand was “acting erratically.” Other times, the “hand was moving slow causing cramping.” Participant 2 described the relationship between control interference and different sensation locations on the prosthetic hand. Specifically, he explained how inadvertent contraction due to stimulation on certain sensation channels associated with the prosthetic aperture sensor and the middle finger sometimes prevented him from opening his hand when he wanted to.

*“I did adjust it (the stimulation intensity) a few times… …The bend (aperture) sensor was the strongest one and that’s the one I kept bringing down. That one and this one (gestures toward left middle finger with left thumb).Which both of those are ones that feel like they have some contraction to them. I mean, I shook the banker’s hand, and umm, ok, hold on, just a second; I can’t, because it wouldn’t let me open up. So I relaxed my arm for a second then it let me open. And a few times I’d get it where both of those were... they would kick in or both of them would kick in, both of ‘em kicking in, it’d wanna (close) (closes left hand slowly as if it were the prosthesis).” (P2)*

Additionally, participant 2 described another event when he was “grabbing” an object but “couldn’t open (his prosthesis) back up.” He mentioned that it would “squeeze a little bit tighter,” until he “reset for a second, and then (it), (would) open.”

Participant 1 described a similar phenomenon saying that he sometimes had “conflict between sensors activating and operating (the) hand.” He commented:

*“And then I was worrying about when we had the shoot (photo shoot), if it was going to make the hand open and close. But it didn’t do that until yesterday. Yesterday, and maybe once the day before, but that was it. What the hell is going on? I don’t remember this.” (P1)*

SENSATION EXPERIENCE

***Location***

Comments about the location of the sensory percepts were coded in this sub-category. In general, participants indicated that the locations of the perceived sensations on the missing hand aligned well with the locations of the sensors on the prosthesis that triggered each sensation. For some locations, the area of the perceived sensations also matched the size of the prosthetic sensors, which were the size of the most distal segment of the fingers.

*“Yeah. It was pretty much right where, right in the pad area. And I know I used to just get the tip, but it was more that whole area there. This one (gestures toward index finger) was that whole area (gestures from distal interphalangeal joint upwards to tip of index).” (P2)*

However, one participant remarked about instances when the perceived location of the sensation did not align accurately with the location of a prosthetic sensor, and indicated that he would prefer for the locations to be more precisely aligned.

*“I guess I would want [the perceived location of the middle finger] more on the pad. It was a little off to the side.” (P1)*

The participants generally referred to a particular prosthetic finger when describing a perceived sensation location, suggesting that the constructs of the prosthesis and the perceived sensation overlapped.

*“Depending on the object, I did hold, I did feel it in these two (points to index and middle of prosthesis).” (P1)*

Given the perceived location, and without visual feedback, both participants could identify which finger of the prosthesis was being touched. Participant 2 described an interaction with his daughter in which she tested his ability to accurately identify the prosthetic finger that she was touching.

*“She’s like “can you feel?” I says “yeah” here, I put my arm back like this, I couldn’t see. I say “you touch” and you know, by which sensation was what I could tell her which finger….. Cause I, I just, I thought about which one, where do I feel the sensation at when she was touching it. Ok, now where do I feel it at now that she’s touching it? I just focused my mind on which one had what sensation and then go from there for the day.” (P2)*

***Timing***

The participants stated that the perceived sensations were synchronous with the visual and auditory feedback they received from observing the prosthesis. When asked about sensation timing, participant 1 commented that he “didn’t feel no delay.” Participant 2 described an experience of picking up a water bottle:

*“Yeah when I grabbed it, it felt like it was pretty much right at the time I did.” (P2)*

The immediacy of sensory feedback allowed participant 2 to shake hands with appropriate force and timing.

*“One of the bankers that I’ve dealt with for 20 years, he come out and seen me there and he wanted to see the new hand. And I shook his hand (extends right arm as if reaching for a hand shake, then motions handshake) and it felt like, when it (squeezes end of stump with left hand) grabbed his hand that’s where I stopped at and that’s when I felt it.” (P2)*

***Intensity***

Comments about the intensity (magnitude) of the sensory percepts were coded in this sub-category. Participants had the most control over this aspect of the sensory experience, since they could adjust their stimulation levels on their own throughout the day. Each location had a preset stimulation level, which participants could adjust on a location-by-location basis. Typically, the participants adjusted the stimulation levels to set a comfortable intensity for each sensory percept at the beginning of each day. Participant 2 commented on his experience with his morning daily adjustments to the sensation associated with prosthetic aperture:

*“I did adjust it a few times. Sometimes I just wasn’t around the house. Out going, and just left it go where it was at. A lot of times it was…The bend sensor was the strongest one and that’s the one I kept bringing down....” (P2)*

Different sensation locations may have had different sensation intensities, and those with stronger intensities sometimes overwhelmed those that felt weaker.

*“Especially, it was even, like I said it was just strong on the bend (aperture sensor), it was just real strong here (points to left thenar eminence using right thumb). And that seemed to overwhelm (points to index and middle pressure sensors using left index). And then the middle finger would come on with the bend (aperture sensor) sometimes… But other than that, it was fine. Like I said, for some reason, I started noticing the pressure along with the vibration.” (P1)*

STABILITY OF SENSATION

***Changes in intensity***

Both participants commented that their perceived sensations tended to get stronger throughout the day. At various points during the day, they reported that they needed to turn down the stimulation levels so that the intensity felt more appropriate.

*“But it seemed like toward the end of the day, it was like, I wanted to turn it down a little more. It seemed like it got stronger.” (P1)*

*“I had to adjust stim [sensory stimulation] a couple of times because it got a little stronger as the day went on” (P2)*

*“But then I was like, wait a minute, this is getting a little stronger….if I turn it down… I turn it down, and then I said man, this thing is getting strong again.” (P1)*

This daily fluctuation in intensity and the stimulation adjustments it necessitated influenced participant 2’s experience with the sensory stimulation system. When asked to describe anything that he “really didn’t like” about the system, he commented:

*“Having to shut it [the stimulator] off and re-calibrate the sensors. It got too strong.” (P2)*

Participant 1 suggested that the daily increases in intensity seemed to be related to the amount of time he was using his prosthesis and receiving sensory stimulation.

*“And it was like, here it’s fine (gestures toward lab, meaning in-lab sessions). But when I had it, and I was using and using it like, I had to keep turning it down cause it was a little too much.” (P1)*

Participant 1 reported that sensations “didn’t fade away” when he held an object or pressed the sensor for a long period of time, instead, “it stayed on.”

Participant 2 stated that he didn’t like holding objects for long periods of time because of changes in the sensation intensity.

*“There were times when I held something for long periods of time, I was like, ok, I gotta switch hands (demonstrates passing an object from prosthetic hand to left hand) now for a little bit. Just because of that and just because I don’t necessarily want to get attenuation or have the chance of it either going away or it getting a little stronger.” (P2)*

Participant 2 commented that the sensations would become less noticeable the longer they were activated.

*“If I, if I think about it (grasps block with prosthetic), for a little while, yes, it’s there. It’s stronger to start off with. After a while, I still notice it, but it’s not as noticeable. I still notice it, but it’s not as noticeable the longer…” (P2)*

***Changes in modality***

The sensation modality appeared to change over the course of the study for Participant 1. The degree to which the sensation felt like tingling decreased or the participant focused less on the tingling component over time.

*“At home, when I turn it down, I seem to more focus on the hand and what was going on other than the tingling. It seemed like I focused more on its holding things. With the pressure component, I didn’t focus on the tingling so much as I did the pressure component. When I picked up stuff, it seems like it just blocked out the tingling. It felt like I was grabbing something.” (P1)*

*“I still felt the tingling, but it wasn’t as strong and sharp. But I still felt the same amount of pressure.” (P1)*

Participant 2, in contrast, stated that the sensation “quality stayed pretty much the same.”

***Changes in location***

Participant 2 experienced some instances where the location of the sensation tied to the thumb sensor would shift during the day. The sensation tended to start out covering both the thumb and the index finger, but by the end of the day, would shift to only cover the thumb without including the index finger.

*“But the thumb and the index, the ones that, I think it was channel 1, the thumb and the index -started out when I’d calibrate it, I could feel it more in the finger there (gestures toward index finger). But then I’d when I’d use it, by hour or two hour wearing it, then it was stronger in the thumb, and then usually didn’t feel it much in the index finger… Cause if I’d press on the thumb (gestures pressing on left thumb with left index finger), yep, just thumb, yep.” (P2)*

He also experienced similar changes in location for the other finger sensors, where the perceived location would shift to match the prosthesis sensor location over the course of the day.

*“The location a lot of times, umm, (gestures toward thumb, webbing and index of left hand using prosthetic to point), one was both of these. And shortly afterwards, using it and everything for a little bit, then it got to where it was just (gestures on thumb) right there in the thumb. Same thing (gesturing on index finger) this one was usually these two (gestures toward index and middle) and it’d get more where it was feeling in this (gestures toward index). This one (gestures toward middle finger) got to where it was feeling just this one (gestures toward middle finger), sometimes it was this one and this one (gestures toward index and thumb), but this one down to about right here (gestures down from index finger, across palm immediately below index finger, into base of palm). And the bend [aperture] sensor (demonstrates hand open/close) it was (gestures around whole median innervated area dorsally – thumb, index, middle and palm associated with these areas) all three of them in the morning when I calibrate. Sometimes later in the day it’d feel like the whole hand (demonstrates whole hand open and close). It’d feel like these two (wiggles ring and pinky fingers) were involved with it too.” (P2)*

***Changes related to arm position***

Participant 1 found that the aperture sensor could be triggered by arm movements, likely reflecting a poor electrical connection for this sensor.

*“And if I pushed on it like this (pushes edge of hand into edge of table), the bend would come on. If I raise my hand, arm, up (raises right arm over head), the bend would come on.” (P1)*

Participant 2 experienced an instance in which one of his sensation channels could not be felt, even at maximal stimulation levels (within safety limits). However, once he made some abrupt arm movements, the sensation was perceived again.

*“Like I said, channel 1 was gone, and then it come back. The next day it wasn’t there at first, but then I went like this (makes a fast motion with right arm across body from shoulder) and moved my arm around some (continues making large motions with right arm from the shoulder) like that, on purpose, and boom, it come back.” (P2)*

He also noticed that the intensity of the sensation appeared to be related to arm position in some cases.

*“That was my thing was to (moves right arm across midline of body from shoulder), a lot of times just to try to move over there. Ok, is it coming back? Ok, yeah, there it is. (moves arm farther across body) Ok, that’s a little strong, let’s move it back (laughs). Move it back (moves right arm so that elbow is below shoulder again), it’s like, ok, do I want to adjust [the stimulation parameters] down? Well, no, because when it’s over here I can barely feel it (right arm held relaxed next to his body). (moves right arm across midline of body so that right elbow is towards sternum) over here, uh, it’s not too strong, but a little stronger than what I want, so then I watch what I do and most of the time I’m just like this (moves both arms in front of body with swishing motions as if doing task directly in front of body), I’m not reaching across like that (reaches right arm across body again with elbow approaching sternum). I reach back like this (moves right arm in extended position posteriorly from shoulder) and sometimes wouldn’t feel anything… (shrugs)” (P2)*

***Consistency of sensation***

Participant 1 felt that the sensations were pretty stable in general, and that he “didn’t have any problems” with sensation stability. However, he also had a malfunction of his aperture sensor, which triggered “very erratic” sensations that “sometimes overwhelmed other areas.”

Participant 2 had a few instances in which he could not find a suprathreshold level of stimulation parameters for some contacts within the safety limits imposed in the stimulator program. In these cases, he “could not feel” the sensations associated with those channels. He also said that “a couple of the channels seemed to come and go” during one of the days of the study.

SYSTEM OPERATIONS

***Mechanical hand interactions***

The pressure sensors were embedded in silicone and covered with a plastic finger cover to make them more water resistant. Participants reported that they sometimes had trouble activating the sensors due to the position of the sensor within the silicone.

*“I mean, the thumb (presses on thumb sensor of prosthetic), I had to squeeze a little harder on some things to get the thumb to work, cause it’s so embedded in the silicone… You know, if it was a softer object, it took a little more squeezing for the thumb to come into play. But harder objects, the thumb came in a lot sooner.” (P1)*

However, the silicone on the fingertips also appeared to provide friction that aided in object grasp.

*“The grippy-ness was better… Yeah, the grip, it was better….. Cause with the other hand (referring to his own prosthesis) I’d have to squeeze more to hold stuff. With this (the sensorized prosthesis) I could squeeze less because of the grippy-ness, I could squeeze less and not overwork it, so to speak. Overwork the hand.” (P1)*

One participant reported that the hand sensors were sometimes triggered without pressing on them. This appeared to be due to the attachment of the silicone to the prosthesis cosmetic glove, which would sometimes pull on the sensors and cause them to activate inadvertently.

*“There’s times, just sitting there, that the thumb will get strong enough where, well, you may see quite a bit where the sensors were triggering off, because when you close the hand it pulls on that cover and that was pulling sensors.” (P2)*

The participant was able to “fiddle with it” or manipulate the sensors with his contralateral intact hand, to eliminate the unintended sensor activation.

*“I know, when I get about like, if it’s about like that (puts left hand in a semi-closed, relaxed position), it wouldn’t fire as much, but when I get like that (closes left hand more until fingertips are about an inch apart) it’d pull enough on that thumb and adjust it, that the outside cover press against there would fire it off and then I’m trying (gestures as if pressing on finger). And sometimes I’d just push on the top (gestures to top of thumb), and it’d go away. And I even took the cover off a few times and it’d still be there and I’d push on the top. And then it would go away, and when I’d release it, it’d come back, so I don’t know…” (P2)*

***Stimulator program***

The stimulation parameter settings returned to preprogrammed levels each time the stimulator turned on. If the participant changed stimulation settings during the day, these changes were not saved by the stimulator, instead the stimulation settings reset to the original preprogrammed levels the next time the stimulator was turned on. This feature was likely not explicitly explained to participants at the outset of the trial, and they were clearly frustrated by the fact that the stimulator “reset itself.” When asked how he felt about the stimulation program, participant 1 commented:

*“Umm I think the only annoying part of it was when it reverted back to… the original state.” (P1)*

When initially turned on, the stimulator also tended to give participants a “jolt” or shock. This was likely due to the offset in the participant’s body ground and the ground of the stimulator battery.

*“I do know that it gives you quite a little jolt when you first turn it on.” (P1)*

Participant 1 frequently wrote about problems with the stimulator “working erratically,” “not powering on,” having “issues powering up,” being “frozen,” shutting off unexpectedly, or “dying.” Both participants experienced issues where the stimulator program would become unresponsive to button press input after being worn for several hours. Because of this, if the participant wanted to change his stimulation levels during the day, he would have to turn off the stimulator in order to do so. “Having to shut it (the stimulator) off and re-calibrate the sensors” was seen as an undesirable aspect of the system.

*“The one thing I didn’t like was the fact that… like you go to hit “Stop Stim” after it’s on for so long, the box (stimulator) won’t stop. So I gotta shut it down, turn it back on, so then I have to recalibrate everything. And couldn’t do it when I was away from home because I didn’t have my sensor block (holds up sensor calibration block with left hand).” (P2)*

For participant 2, at his request, the sensation associated with the aperture sensor would cease if there was no change in input on the sensor for 30 seconds. The participant liked having this feature because it enabled him to keep his prosthesis in a partially closed position without continually receiving stimulation.

*“EG (researcher) did set it so that after it’s 30 seconds it would, the bend (aperture) sensor would shut off (holds left hand in a partially closed position to demonstrate). Cause, like I told her, [my prosthetist], a lot of the newer prosthetics, are coming out. No body walks around with their hand like this (opens prosthetic hand all the way). I don’t either. I’ll always put it about like that (closes hand partially) so that it looks more normal and then they’re setting the newer prosthetics so that after so long of no use, it goes to a home. So that it looks more normal. So she did add that to it, which was great, because after 30 seconds it would shut off.” (P2)*

***Connectors and cables***

The participants’ percutaneous leads were protected from snagging or getting pulled due to tethering by a magnetic quick-disconnect cable. Participants reported that the magnetic quick-disconnect cable was easy to connect or “pop right back on.” It also functioned well in protecting the leads from pulling. Participant 1 described an experience where he was walking down a crowded hallway and the cabling became snagged:

*“And I was walking around with this (gestures to cable at elbow) cause it was catching on the door handles (points to door handle). Because people were walking real close, I had to get close to the wall. I was like ‘god’ (gestures as if caught wires at elbow on something while trying to keep walking) (gestures as if trying to untangle wires). So other than that… that’s why I wore the long sleeve shirts all the time so I wouldn’t have that issue.” (P1)*

However, the quick-disconnect cable also became disconnected due to movement through their normal range of motion or during reaching. Participant 1 stated that he’d prefer for the “magnets to be a little bit stronger, so it doesn’t break away from reaching so much.”

*“And this one (gestures to magnetic quick-disconnect connector) only came off a couple times, where it would either catch on the shirt or on the skin. It seemed like when the skin gets wet, that stuff, the wrapping (talking about the cable coating) gets real sss (gestures as if tugging back and forth with left hand) it wants to grab it. And it pulls (gestures right arm across body). Sometimes if this (the cable) falls down (gestures to right shoulder with left hand) behind the shoulder, and I go to reach for something (reaches with right arm across body) real quick, and it’s tight, it popped off (gestures as if cable disconnecting).” (P1)*

Participant 2 reported similar instances where his magnetic quick-disconnect cable was pulled apart during usage.

*“Just a couple times it got disconnected and that’s more so because holding a little one (rubs right shoulder with left hand) or something and they (rubs shoulder) drag their hand across and it pop loose and I wouldn’t notice until I’d go to grab. Oh, ok, no sensation. Start checking (mimes checking cables on body).” (P2)*

Participant 2 disliked having to wear the stimulator in a fanny pack at his waist and became frustrated with his cables. He described having to “get used to the wires and fanny pack.”

“*Fanny pack get in the way sometimes. I’m not much for wearing a fanny pack. Like I told Emily, (picks up cabling) I taped a few pieces of tape on cables together because I got tired of doing one cable, then try to do the other cable, and adjust them both, and tape them both down.” (P2)*

***Changing stimulation parameters***

The participants described their methods for adjusting their stimulation levels each day. They were able to change the pulse width between 1-255 µs, pulse amplitude between 0.1 and 1.0 mA, and stimulation pattern between four different patterns. Participant 1 said that he tended to only change pulse width of the stimulation, and only rarely adjusted the pulse amplitude or stimulation pattern.

*“I think I messed with the pattern once, and I didn’t like the pulsing one, and I just left it on pattern 1 the whole time. I just reverted back to that one. The other ones didn’t seem like too much of a difference to me, where it mattered, so I just left it at 1.” (P1)*

In general, the participants found adjusting the stimulation parameters to be an easy process.

*“Once I got in the menu, it was one, two, three, to be honest with you. The more, you know, like yesterday I zipped right through it. And it was fine. Changing the stim and everything was very easy. I couldn’t ask for anything easier to be honest with you.” (P1)*

Both participants described wanting to adjust the stimulation levels because it “got too strong” in intensity or “got stronger as the day went on.” Participant 1 tended to only adjust his stimulation levels at the beginning of the day, and seldom changed it throughout the day. When asked whether he had to change his stimulation levels during the day, participant 2 commented:

*I did some days and some days I was just, I was out on the go, so I just left it where it was at. (P2)*

When he did need to change the stimulation settings during the day, participant 2 commented that this was typically most needed for the sensation associated with his aperture sensor, because the sensory stimulation on this channel tended to evoke muscle contraction, and was “the strongest one.”

*But that one, in the mornings, every morning, it [the bend sensor] was so strong that it would just automatically (holds left hand up), it was like (quickly tightens and closes left hand), mmmm (grunts). Ok. We’re going from 7 down to 6, now from 225 down to 125 (referring to different stimulation parameters) – That’s better. That’s better. And then by the end of the day, it was so strong that I might bump it down to 75 or a little lower even.” (P2)*
