# Supplementary material for: The benefits of sensation on the experience of a hand: A qualitative case series
Source: PLoS One. 2019 Jan 31;14(1):e0211469. doi: 10.1371/journal.pone.0211469 (PMC6355013; doi:10.1371/journal.pone.0211469)
Supplement: S2 Appendix — (DOCX) [file pone.0211469.s002.docx]

**S2 Appendix.** Survey questions and interview question examples.

**Survey Questions**

The following are questions from the free response sections of the surveys. The first question was asked during all in-lab testing sessions. The second and fourth questions were asked daily in all study stages as part of the daily diary. The third question was asked daily in the sensory-enabled stage of the study as part of the daily diary.

- Please describe the sensation of using the prosthetic hand.
- Please write any comments you have about your experience with the provided prosthesis today.
- Please write any comments you have about your experience with the sensory stimulation today.
- Please write any other comments about today. For any day that you did not wear the provided prosthesis, please provide a note.

**Interview Question Examples**

A selection of interview questions are provided below. These questions were asked after the week of home use with sensation enabled.

- How would you describe the experience of having the sensory restoration system?
- How would you describe the week (of having sensation enabled at home) and your experience?
- Did the sensory feedback make a difference?
- How would you explain the difference of having the sensation vs not having the sensation?
- What did you think about touching with the prosthesis around your house?
- Did you find that you needed to change the stimulation levels every day?
- Did you adjust the stimulation parameters to the same thing each day?
- What frequency did you calibrate the sensors?
- When you were using the system, were you thinking about it?
- Were you paying more attention to the prosthesis with sensation?
- Did you feel like you were having to focus on what you were doing with the prosthesis?
- Did you have to think about using the hand?
- How satisfied were you with how the pressure sensors turned on in response to objects?
- Did you feel like, when you touched things, that there was a noticeable delay?
- What did the sensation feel like?
- What do you think about the type of sensation that you were getting?
- Did the sensation quality change?
- Did you feel like the sensation shifted to match where the sensor actually was?
- Was the sensation the same across all of your locations?
- What was the sensation intensity?
- Did the sensation change in intensity?
- Did you ever feel like the sensation went away?
- When you held something constantly, did the intensity feel the same the whole time?
- Did the sensation tell you something about what you were holding?
- Did you find the sensory information to be useful?
- Was the sensation helpful in operating or using your prosthesis?
- Did you feel like the response time of the sensation in response to touch was useful for you?
- What was the best thing about the system?
- Did you have any problems with the system?
- Did you like having the system?
- Was there any functionality in the system that you wished you had?
- Is there anything that you either really liked or really didn’t like about the system?
- Do you think you were getting interference with your myoelectric control?
- Did you use the system all day long?
- Did you have any problems with the connectors?
- Did you think it was useful to have the break away connector?
- What were the challenges about having all the external cabling and hardware?
- How difficult was it for you to move your arm around in space?
- How satisfied were you with the battery life?
- How happy were you with the variety of activities that could be done while wearing the system?
- Did the fingertip sensors change any of the interactions of the prosthesis or doing anything?
- Did you feel more confident or less confident with sensation?
- Did the prosthesis feel like it was your hand?
- How did you feel about the prosthesis when you had sensation?
- Did the sensation change the way you thought about your prosthesis at all?
- Did you change how you used the prosthetic?
- Is the sensory stimulation more noticeable than sensation in the other hand?
- How did the sensation feel relative to your other hand?
- Would you use the system full time if you were able to?
- Did the benefits of having sensation make it worthwhile to put on the system?
- Would you want the sensory restoration system to replace your own prosthesis?
- Did the sensation annoy you through the day?
- Did you ever find that you wanted to squeeze on things just to activate sensory stimulation?
- Did you prefer the sensory feedback?
